# Pericardial decompression syndrome

**DOI:** 10.5339/qmj.2024.qitc.30

**Published:** 2024-03-26

**Authors:** Um I Rubab, Shaikh Nissar

**Affiliations:** SICU – HGH, Doha, Qatar Email: uzafar@hamad.qa

**Keywords:** Hemodynamics, pulmonary edema, pericardial effusion, pericardial decompression, surgical drainage

## Background

The massive amount of pericardial fluid accumulation with or without cardiac tamponade needs drainage. Following the drainage procedure, there may be hemodynamic deterioration in the cardiac status of the patient, leading to transient left ventricular dysfunction and subsequent pulmonary edema. This phenomenon is called pericardial decompression syndrome (PDS).^1^ PDS is a potentially life-threatening complication associated with drainage of pericardial fluid either surgically or via image-guided drainage. It is often underreported, poorly understood, and often overlooked. It is an acute emergency, and intensivists, anesthetists, and surgeons should be aware of this rare clinical condition.

## Case

**Figure 1.**
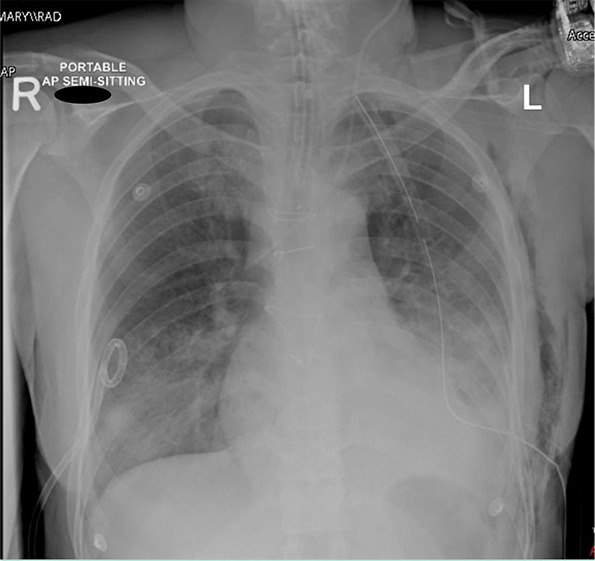
X-ray showing pulmonary edema.

Following open repair of the right subclavian artery pseudo aneurysm in a young patient, significant pericardial effusion occurred, necessitating surgical drainage that resulted in the removal of 1500 ml of transudate pericardial fluid. In the postoperative period, despite initial improvement and extubation, the patient experienced a decline, manifesting as acute pulmonary edema (Figure 1). As shown in Table 1, the patient was vitally unstable. This led to the need for reintubation and the initiation of vasopressor and diuretic therapy, ultimately diagnosed as PDS. Upon admission to the intensive care unit, the patient received supportive therapy and showed improvement within 48 hours. This progress enabled successful weaning of vasopressors and extubation of the trachea.

## Conclusion

Patients who undergo either surgical or image-guided drainage for substantial pericardial effusion may experience the development of PDS. Early detection and management in the intensive care unit are crucial.

## Conflict of Interest

Both authors have no conflict of interest.

## Figures and Tables

**Table 1.** Vital parameters.

**HR**	**RR**	**SpO_2_**	**BP**
130	30	80	89/48
